# Return of the chickpea zombies: how *Ascochyta rabiei* overcomes cell-wall defenses to cause disease

**DOI:** 10.1093/plcell/koad008

**Published:** 2023-01-19

**Authors:** Bradley Laflamme

**Affiliations:** Assistant Features Editor, The Plant Cell, American Society of Plant Biologists, USA; Department of Molecular Genetics, University of Toronto, Toronto, ON M5S 1A1, Canada

Plant pathogens exhibit biotrophic and/or necrotrophic lifestyles. Biotrophs, which require a living host to sustain their infection cycle, often carefully modulate host physiology early in an infection to produce an appropriate niche for themselves (Xin et al., 2016). In contrast, the necrotrophs, though very much alive, often evoke images of the undead zombies of horror movies: rapidly breaking down any barricades to kill and feed on dead host tissue. The cell wall is perhaps the most important fortification that plants use to defend against pathogens (Lee et al., 2019). Whether the host adequately reinforces this barrier or the pathogen breaks through is a reliable predictor of how this horror movie will end: either in favor of our crops or in favor of the zombies. Despite cell-wall degradation being a unifying trait of diverse necrotrophs, we have a limited understanding of their scope of tools to dismantle the cell wall and establish their feast.

In this issue, **Shreenivas Kumar Singh and colleagues** (Singh et al., 2023) uncover a novel approach to suppressing cell-wall-mediated defense in *Ascochyta rabiei*, a necrotrophic fungal pathogen of chickpea (*Cicer arietinum*). Through an investigation of the predicted secretome of *A. rabiei*, the group identified ArPEC25, an effector protein that was highly up-regulated during infection. Using reverse genetics, transcriptomics, and microscopy, the group confirmed that ArPEC25 localizes to host nuclei and is essential for virulence in chickpea. Given that most pathogen effectors are individually dispensable (Arroyo-Velez et al., 2020), the requirement of ArPEC25 for virulence suggested that its planta nuclear target is key to establishing infection (see Figure).

**Figure:**
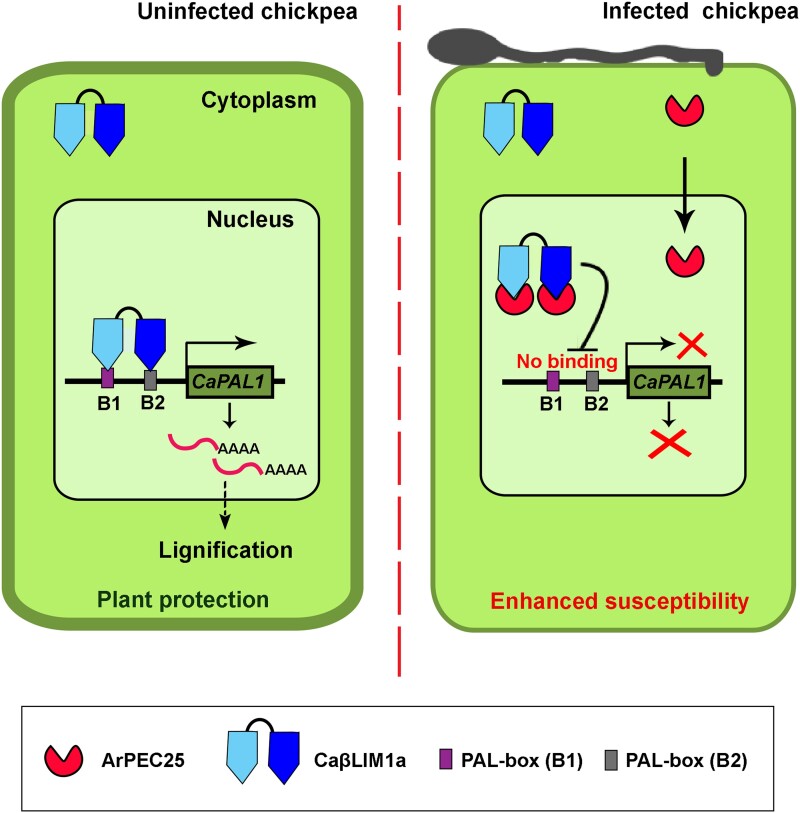
Schematic of CaβLIM1a activity with (right) or without (left) *A. rabiei* infection. In uninfected chickpeas, CaβLIM1a binds to the *PAL1* promoter and activates lignification. Upon infection, ArPEC25 binds to CaβLIM1a and prevents its interaction with *PAL1* promoters, thereby stalling lignification. Reprinted from Singh et al. (2023), Figure 9.

To explore how ArPEC25 modulates chickpea physiology, Singh et al., performed a yeast two-hybrid screen against a cDNA library isolated from *A. rabiei-*infected chickpea. CaβLIM1a, a LIM-family transcription factor, was among the most promising hits with ArPEC25. The interaction between ArPEC25 and CaβLIM1a was confirmed with in vitro pull-downs and two in planta methods with *Nicotiana benthamiana* (bimolecular fluorescence complementation and Förster resonance energy transfer acceptor photobleaching). Since LIM transcription factors have been shown to activate the phenylpropanoid biosynthetic pathway—a key pathway in the production of the biopolymer lignin, which is required for cell-wall fortification under pathogen stress (Zhang and Liu, 2015; Lee et al., 2019)—the group explored whether CaβLIM1a could stimulate this pathway in chickpea. Both electrophoretic mobility shift and yeast one-hybrid assays were used to show that CaβLIM1a binds to the promoter of the chickpea gene encoding phenylalanine ammonia-lyase1 (CaPAL1), a rate-limiting enzyme in lignin biosynthesis. Further investigation with a dual-luciferase reporter system confirmed that *CaPAL1* can be activated by CaβLIM1a.

Significantly, the *CaPAL1*-promoter-binding activity of CaβLIM1a was decreased in the presence of ArPEC25. Furthermore, metabolic profiling of chickpea plants infected with either wild-type or *Δarpec25 A. rabiei* confirmed that lignin biosynthesis was only interrupted in the presence of ArPEC25. Thus, the authors established that the interruption of phenylpropanoid biosynthesis by ArPEC25 is a key to interrupting lignification of the cell wall. Hence, without ArPEC25, the pathogen fails to overcome the plant's fortifications and the horror movie ends prematurely.

The zombies of horror movies are often depicted as braindead, but not *A. rabiei*: Singh et al., because it has already depleted your lumber supply. Now whatever you do, *don’t turn around!* That’s the mistake everyone always makes in these movies.
